# Dandelion sterol improves diabetes mellitus–induced renal injury in in vitro and in vivo study

**DOI:** 10.1002/fsn3.2491

**Published:** 2021-07-28

**Authors:** Lin Tian, Peng Fu, Min Zhou, Jiping Qi

**Affiliations:** ^1^ Department of Pathology The First Affiliated Hospital of Harbin Medical University Harbin China; ^2^ Department of Nuclear Medicine The First Affiliated Hospital of Harbin Medical University Harbin China

**Keywords:** Dandelion sterol, DN, miR‐140‐5p, NF‐κB(p65), Renal injury, TLR4

## Abstract

The purpose of our research was to evaluate Dandelion sterol's treatment effects on diabetes mellitus–induced renal injury in in vitro and in vivo study. The rats were divided into five groups as normal control (Ctrl), diabetic nephropathy model (Model), Dandelion sterol low‐dose treated (Dan‐Low), Dandelion sterol middle‐dose treated (Dan‐Middle), and Dandelion sterol high‐dose treated (Dan‐High). Measuring serum TNF‐α, IL‐1β, and IL‐6 concentrations by Elisa assay, evaluate kidney pathology by HE staining, kidney cell apoptosis of TUNEL, TLR4, and NF‐κB(p65) proteins expression by IHC assay, and relative gene expressions by RT‐qPCR assay. In the following step, using HK‐2 treated with high glucose to model DN cell model to discuss the relative mechanisms, evaluate TNF‐α, IL‐1β, and IL‐6 concentrations by Elisa assay, evaluate cell apoptosis by flow cytometry, evaluate TLR4 and NF‐κB(p65) proteins expression by WB assay, relative gene expression by RT‐qPCR assay, and NF‐κB(p65) nuclear volume by cellular immunofluorescence. Compared with Ctrl group, TNF‐α, IL‐1β, and IL‐6 concentrations and apoptosis cell number were significantly increased, TLR4/NF‐κB(p65) pathway was significantly stimulated in Model rats and cell groups. With Dan supplement, the diabetic‐induced renal injury was significantly improved (*p* < .05, respectively). By cell experiment, Dan improved cell apoptosis and inflammatory factors via miR‐140‐5p. Dan improved diabetes mellitus–induced renal injury via regulation of miR‐140‐5p/TLR4 axis in in vitro and in vivo study.

## Graphical abstract

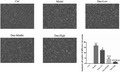

## 1 INTRODUCTION

As suggested by a large number of studies, renal tubular injury plays an important role in the occurrence and development of diabetic nephropathy (DN) (Eriguchi et al., 2018; Han et al., 2018) but it fails to completely explain the pathogenesis. Meanwhile, there were no effective drugs to treat DN in clinical. Toll‐like receptor 4 (TLR4), a pattern recognition receptor belonging to the TLR family, was suggested to be closely related to immune and inflammatory responses (Hughes et al., 2019; Lin et al., 2012). In addition, it has been proven that TLR4 can promote the occurrence and development of renal tubulointerstitial injury in DN (Jheng et al., 2015). MicroRNA (miRNA), an endogenous noncoding single‐stranded RNA of approximately 22 nucleotides in length, has been reported to play crucial roles in regulating a variety of cellular biological processes, such as cell differentiation, proliferation, and apoptosis, through the degradation or translational inhibition of target mRNA via completely or incompletely complementary pairing with the target mRNA (He & Hannon, 2004; Kim et al., 2009). Moreover, recent studies revealed that miRNA plays an important role in the occurrence and treatment of diabetes mellitus (DM) and various DM‐related complications, especially DN (Banerjee et al., 2017; Conserva et al., 2013; Kantharidis et al., 2011). Furthermore, it has been demonstrated that miR‐140‐5p, a newly discovered miRNA, can inhibit inflammation via targeted regulation of the TLR4/NF‐κB (p65) signaling pathway (Papathanasiou et al., 2020; Su et al., 2020) and improve apoptosis caused by inflammation (Huang et al., 2019; Sun et al., 2017).

Dandelion, as a traditional Chinese herbal medicine, had a wide range of pharmacological activities (Alzoman et al., 2019). Previous studies suggested that Dandelion sterol, a dandelion sterol, has antioxidant, antiinflammatory, antibacterial, antitumor, and other pharmacological effects (Takasaki et al., 1999; Yousefi Ghale‐Salimi et al., 2018). However, the role of Dandelion sterol in DN and the related mechanisms have not been reported. Therefore, in this study, the effects of different doses of Dandelion sterol on diabetic renal injury and the influence of miR‐140‐5p were observed through in vivo and in vitro experiments. Then, in vitro cell experiments were performed to investigate whether Dandelion sterol could regulate TLR4/NF‐κB (p65) signaling through miR‐140‐5p to improve diabetic renal injury.

## 2 MATERIALS AND METHODS

### 2.1 Animals and cells

Healthy SPF‐grade *SD* rats (age, 4 weeks old; gender, male; and weight, 130–140 g) were provided by Guangdong Medical Laboratory Animal Center (Animal Licence No. SCXK [Yue] 2013–0002). HK‐2 human renal tubular epithelial cells were purchased from Nanjing KeyGEN Biotechnology Co., Ltd.

### 2.2 Drugs and reagents

Dandelion sterol (99.9% purity, Chengdu Rifensi Biotechnology Co., Ltd.), streptozotocin (STZ; specification: 1 g per bottle; batch number: 8110047), TLR4 antibody (Abcam, UK), NF‐κB (p65) antibody (Abcam, UK), GAPDH antibody (Abcam, UK), TRIzol (Invitrogen, USA), a PrimeScript™ RT reagent kit (Takara, Japan), SYBR Premix Ex Taq™ (TIIRNaseH Plus, Takara), a TUNEL detection kit (Roche, USA), miR‐inhibitor‐NC (Nanjing KeyGEN Biotechnology Co., Ltd), and miR‐140‐5p inhibitor (Nanjing KeyGEN Biotechnology Co., Ltd) were obtained from commercial vendors.

### 2.3 Instruments

A Model 680 enzyme labeling instrument (Bio‐Rad Laboratories, Inc., USA), BX51 optical microscope (Olympus Corporation, Japan), and WD‐9413B gel imaging system (Beijing Liuyi Instrument Factory) were used in the analyses.

### 2.4 Establishment of the DN rat model

Rats were fed a high‐fat diet for 4 weeks, fasted for 12 hr, and subjected to an intraperitoneal injection of 35 mg/kg STZ solution to prepare the DN model (Shen et al., 2020). After 72 hr, the 2‐hr postglucose load plasma glucose (PG) levels of rats were measured, and the successful establishment of the DN model was verified by PG levels >11.1 mmol/L.

### 2.5 Animal grouping and administration method

After the DN model was successfully established, the rats were randomly divided into five groups (*n* = 12 each) according to body weight as follows: normal control (Ctrl), Model, low‐dose Dandelion sterol (Dan‐L), medium‐dose Dandelion sterol (Dan‐M), and high‐dose Dandelion sterol (Dan‐H) groups. Additionally, rats fed a normal diet (*n* = 12) were used as the normal group.

The rats in the Ctrl and Model groups were administered an isodose of 0.9% NaCl intragastrically. Rats in the control group were additionally administered rosiglitazone (3 mg/kg•day). Rats in the Dan‐L, Dan‐M, and Dan‐H groups were administered Dandelion sterol (10, 20, and 100 mg/kg•day, respectively). After 8 weeks of intragastric administration in each group, 24‐hr urine samples were collected using metabolic cages, and tail vein and orbital blood samples were collected from the fasting rats. The animals were then sacrificed, followed by removal of the kidneys. Residual blood was removed from the organs via lavage with precooled normal saline, and the kidneys were dried using filter paper for further examination. This study was approved by the ethics committee of First Affiliated Hospital of Harbin Medical University (approval No. 2018–12–036). The entire experimental process followed the “Regulations on the Administration of Laboratory Animals” formulated by the Science and Technology Commission of the People's Republic of China, and the “Guiding Opinions on the Good Treatment of Laboratory Animals” issued by the Ministry of Science and Technology of the People's Republic of China.

### 2.6 Cell culture

HK‐2 cells were cultured in keratinocyte SFM culture medium (containing 0.05 g/L BPE, 5 μg/L EGF, 0.9 mmol/L CaCl2, and 5.5 mmol/L D‐(+)‐glucose) in an incubator (37°C and 5% CO2), followed by digestion with 0.05% pancreatin, and passage at a ratio of 1:3 every 5 days. The cells subcultured in 5.5 mmol/L glucose were used as the normal control group (Ctrl group), and those subcultured in 30 mmol/L glucose were used as DN model group (Model group). Cells subcultured in 30 mmol/L glucose plus 10, 20, or 100 mg/L Dandelion sterol served as the low‐ (Dan‐L), middle‐ (Dan), and high‐concentration (Dan‐H) experimental groups, respectively.

### 2.7 Measurements of fasting plasma glucose (FPG), urinary albumin/creatinine ratio (UACR), body mass, and kidney index (Ki) in each group

FPG levels were detected using blood samples collected from the tail veins of rats, and 24‐hr urine samples of rats were collected using metabolic cages. Urinary albumin and creatinine were measured using the biuret test and used to calculate UACR. Rats were weighed after sacrifice to determine body mass. The excised kidneys of rats were weighed, and Ki was calculated as follows: Ki = rat kidney weight/rat body mass.

### 2.8 Detection of serum inflammatory cytokines in each group

The orbital blood samples of rats and culture medium were taken for the separation of serum and culture medium supernatant via centrifugation at 3,000 *g* for 5 min, and the obtained supernatant was analyzed using ELISA according to the manufacturer's instructions. The absorbance values in each group were measured at a wavelength of 450 nm using an ELISA reader, and the standard curve was obtained. The serum concentrations of TNF‐α, IL‐1β, and IL‐6 were calculated for each group.

### 2.9 Pathomorphological examination of renal tissues in each group

The right renal tissues were fixed with 10% formaldehyde, dehydrated using an ethanol gradient, embedded in paraffin, and serially sectioned at a thickness of 4–5 μm. The prepared serial sections were subjected to H&E staining, followed by observation under an optical microscope.

### 2.10 TUNEL assay

The paraffin‐embedded tissue sections were subjected to conventional dewaxing, hydration, soaking in 3% H2O2 methanol solution, and incubation at room temperature for 30 min. Samples were then sealed with peroxidase and washed. Proteinase K solution was added in a drop‐wise manner, and samples were incubated at 37°C, returned to room temperature, and washed. Samples were then incubated with 0.1% Triton X‐100 and 1% sodium citrate solution, and returned to room temperature and washed. Next, the reaction mixture was added in a drop‐wise manner, followed by incubation at 37°C for 90 min, washing, blocking with normal goat serum, and incubation at room temperature for 20 min. After discarding the serum, transforming agent solution was added in a drop‐wise manner, and samples were incubated at 37°C for 30 min, permitted to recover to room temperature, and washed. The reaction was terminated by washing in water when positive nucleus staining was brownish yellow under the light microscope. The number of positively stained apoptotic cells was counted in each group.

### 2.11 TLR4 and NF‐κB (p65) protein levels in rat renal tissues

The paraffin‐embedded sections of right renal tissues from rats in each group were subjected to conventional xylene dewaxing, soaked in a graded ethanol series for 5 min, and rinsed with double‐distilled water for 5 min to remove ethanol. After antigen retrieval, samples were incubated with deionized water for 10 min, blocked with BSA for 15 min, and incubated overnight with rabbit anti‐TLR4 antibody and NF‐κB (p65) primary antibodies at 4°C. Samples were subsequently warmed at 37°C for 1 hr and incubated with the corresponding secondary antibodies at room temperature for 15 min. Ten fields of vision in the renal cortical area were randomly selected under the microscope (magnification: ×200), followed by observation using the Image Pro Plus 6.0 image analysis system. The relative expression of TLR4 and NF‐κB (p65) was expressed as IOD values.

### 2.12 Detection of apoptosis via flow cytometry

After treatment for 48 hr as previously described, cells were washed with PBS, digested with trypsin, centrifuged, and then washed twice with PBS. After adding 250 μl of buffer solution to each tube, the cell concentration was adjusted to 1.0 × 106/ml after cell counting, and 100 μl of each cell suspension was aliquoted into flow tubes and mixed with Annexin V‐FITC (150 mg/L) and propidium iodide (120 mg/L). The mixture was incubated in the dark at room temperature for 15 min. PBS was added to wash the cells, and flow cytometry (FACS) was used to detect apoptosis.

### 2.13 Western blot (WB) analysis

After treatment for 48 hr as previously described, HK‐2 cells were washed three times with PBS and lysed using RIPA lysate, and the total protein was extracted. The protein concentration was determined using the BCA method, and the remaining proteins were heat denatured by adding 6× loading buffer. The denatured proteins (20 μg/well) were separated via sodium dodecyl sulfate‐polyacrylamide gel electrophoresis (SDS‐PAGE), followed by transfer to a membrane using a voltage of 110 V. The membranes were blocked in 5% skim milk powder for 1 hr, followed by three washes with TBST for 10 min each. Incubation with specific antibodies was performed overnight at 4°C. The membranes were washed three times with TBST for 10 min each on the next day, and the corresponding secondary antibodies labeled with horseradish peroxidase were added, followed by incubation on a shaking table for 1 hr at a slow speed at room temperature. After three washes with TBST for 10 min each, ECL development solution was added, and protein levels were determined using a chemiluminescence fluorescence imaging system (Bio‐Rad). The relative protein expression in each group was calculated using GAPDH as an internal reference.

### 2.14 RT‐qPCR detection

After being fully ground, the collected tissues and cells were mixed with TRIzol solution, and the total RNA in tissues and cells was separated and extracted according to the instructions of the TRIzol kit. After first‐strand cDNA was synthesized by reverse transcription, miR‐140‐5p (U6 as the internal reference), TLR4 (GAPDH as the internal reference), and NF‐κB (p65) (GAPDH as the internal reference) expression was detected using qRT‐PCR. Based on the operating instructions of the kit, primer sequences were configured as follows: miR‐140‐5p, forward, 5′‐ACACTCCAGCTGGGAGGCGGGGCGCCGCGGGA‐3′, and reverse, 5′‐CTCAACTGGTGTCGTGGA‐3′; TLR4, forward, 5′‐AGACATCCAAAGGAATACTGCAA‐3′, and reverse, 5′‐GCCTTCATGTCTATAGGTGATGC‐3′; NF‐κB (p65), forward, 5′‐GAGAGCCCTTGCATCCTTTA‐3′, and reverse, 5′‐CTTCCCTTTGGTCTTTCTGT‐3′; GAPDH, forward, 5′‐GAGTCAACGGATTTGGTCGT‐3′, and reverse, 5′‐GAGTCAACGGATTTGGTCGT‐3′; and U6, forward, 5′‐GCTTCGGCAGCACATATACTAAAAT‐3′, and reverse, 5′‐CGCTTCACGAATTTGCGTGTCAT‐3′.

Primers were synthesized by Sangon Biotech (Shanghai) Co., Ltd. The reaction conditions were as follows: predenaturation at 95°C for 30 s; 40 cycles of 95°C for 5 s, and 60°C for 20 s; and dissociation curve analysis at 95°C for 1 s, 65°C for 15 s, and 95°C for 5 s. After the reaction, the amplification and dissociation curves were confirmed.

### 2.15 Cellular immunofluorescence

After 48 hr of treatment as described previously, the supernatant of cells was discarded when the confluence reached approximately 80%. Cells were washed twice with PBS and then fixed in 4% polymethylmethacrylate for 20 min, followed by three washes with PBS, and blocked with 5% skimmed milk powder for 2 hr. The glass was carefully removed using a deterrent and placed on the glass slide, followed by three washes with PBS (10 min each to remove the residues as much as possible). A mouse anti‐human NF‐κB (p65) antibody (dilution: 1:40 with skimmed milk) was added, cells were incubated at 4°C overnight, and then washed three times with PBS on the next day. FITC‐labeled goat anti‐mouse IgG (diluted 1:100 in PBS) was added, and cells were incubated for 2 hr, followed by three washes with PBS. The glass slide was covered with a cover glass, bifocal observation was performed using a confocal microscope, and cells were photographed for further use. All experiments were repeated three times. The rate of NF‐κB (p65) protein transportation to the nucleus was examined using the Image Pro Plus 6.0 image analysis system.

### 2.16 Statistical analysis

SPSS 17.0 software was used for statistical analysis. Normally distributed data were expressed as the mean ± *SD*, and nonnormally distributed data were expressed as the median (interquartile range). One‐way ANOVA was used for multiple comparisons of data, and the LSD method was applied for comparisons between two groups for normally distributed data. Indices not conforming to a normal distribution were subjected to statistical analysis after logarithmic transformation, and the χ2 test was used for enumerated data. Multisample comparisons among the groups were performed via one‐way ANOVA, and an independent samples *t* test was used for two‐sample comparisons. *p* < .05 indicated a significant difference.

## 3 RESULTS

### 3.1 Comparisons of FPG, UACR, body mass, and Ki among the groups

As shown in Table 1, compared with the results in the Ctrl group, FPG levels, UACR, and Ki were significantly elevated in the Model, Dan‐L, Dan‐M, and Dan‐H groups, whereas body mass was significantly decreased (*p* < .05, respectively). FPG levels, UACR, and Ki were significantly higher in the Model group than in the Dan‐L, Dan‐M, and Dan‐H groups (*p* < .05, respectively).

**TABLE 1:** Comparisons of FPG, UACR, body mass, and Ki among the groups

Group	FPG (mmol/L)	UACR(mg/g)	Body weight (g)	KI
Ctrl	7.05 ± 1.35	3.59 ± 1.89	433.41 ± 28.89	0.24 ± 0.01
Model	24.05 ± 4.11^a^	87.85 ± 10.86^a^	238.03 ± 25.86^a^	0.42 ± 0.09^a^
Dan‐Low	17.31 ± 4.32^a^, ^b^	25.34 ± 7.91^a^, ^b^	260.00 ± 26.49^a^	0.34 ± 0.05^a^, ^b^
Dan‐Middle	16.56 ± 5.61^a^, ^b^	24.94 ± 6.55^a^, ^b^	249.66 ± 27.59^a^	0.33 ± 0.06^a^, ^b^
Dan‐High	15.78 ± 8.05^a^, ^b^	24.59 ± 5.98^a^, ^b^	238.73 ± 30.00^a^	0.32 ± 0.07^a^, ^b^

^a^ *p* < .05, compared with Ctrl group.

^b^ *p* < .05, compared with Model group.

### 3.2 Pathomorphological changes in the renal tissues of rats in each group

**FIGURE 1:**
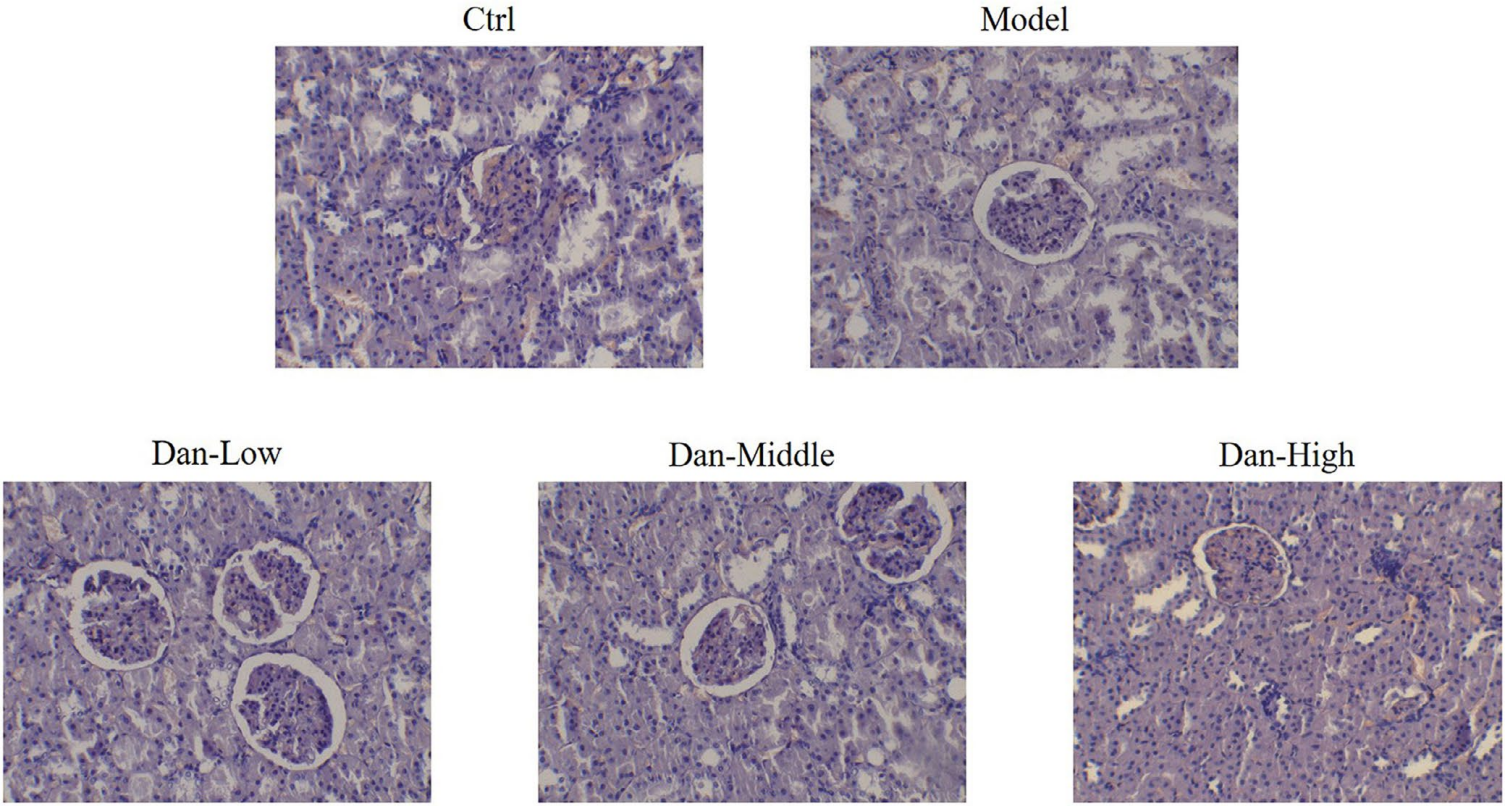
Pathomorphological changes in the renal tissues of rats in each group by HE staining (200×). Ctrl: The rats were treated normally; Model: The rats were given DN model treatment; Dan‐Low: DN model rats treated with 10 mg/kg•day Dan; Dan‐Middle: DN model rats treated with 20 mg/kg•day Dan; Dan‐High: DN model rats treated with 100 mg/kg•day Dan

In the Ctrl group, glomeruli and renal tubules have a normal morphological structure, and there were no significant changes in the glomerular volume and cell number. The capillary and balloon lumina were visible, the glomerular mesangial matrix was intact, and no thickening of the basement membrane was evident. Renal tubular epithelial cells displayed no obvious degeneration and necrosis, and there was no tubular type in the lumen. In the Model group, the glomerular volume was increased compared with that in the Ctrl group, and some renal tubules exhibited slightly or moderately enlarged lumina. Renal tubular epithelial cells exhibited vacuole‐like degeneration and visible inflammatory cell infiltration. Compared with the Model group findings, the pathological damage in renal tubules and glomeruli was alleviated in the Dan‐L, Dan‐M, and Dan‐H groups, as shown in Figure 1.

### 3.3 Comparisons in serum levels of TNF‐α, IL‐1β, and IL‐6 among the groups

The serum concentration levels of TNF‐α, IL‐1β, and IL‐6 were significantly higher in the Model group than in the Ctrl group (*p* < .05, respectively). Meanwhile, the serum levels of these cytokines were significantly lower in all three groups of Dandelion sterol‐treated rats than in the Model group (*p* < .05, respectively). Relevant data are summarized in Table 2.

**TABLE 2:** TNF‐α, IL‐1β, and IL‐6 concentrations in serum

Group	TNF‐α(pg/ml)	IL−1β(pg/ml)	IL−6(pg/ml)
Ctrl	86.10 ± 6.61	248.65 ± 38.03	145.58 ± 16.54
Model	134.40 ± 27.87^a^	530.68 ± 84.05^a^	254.62 ± 25.46^a^
Dan‐Low	107.19 ± 19.82^a^, ^b^	344.59 ± 19.19^a^, ^b^	200.87 ± 46.24^a^, ^b^
Dan‐Middle	95.64 ± 19.77^a^, ^b^	290.66 ± 20.59^a^, ^b^	186.65 ± 30.54^a^, ^b^
Dan‐High	85.26 ± 13.69^a^, ^b^	269.93 ± 36.98	172.30 ± 27.88^a^, ^b^

^a^ *p* < .05, compared with Ctrl group.

^b^ *p* < .05, compared with Model group.

### 3.4 The apoptosis cell number of different rat groups by TUNEL assay

**FIGURE 2:**
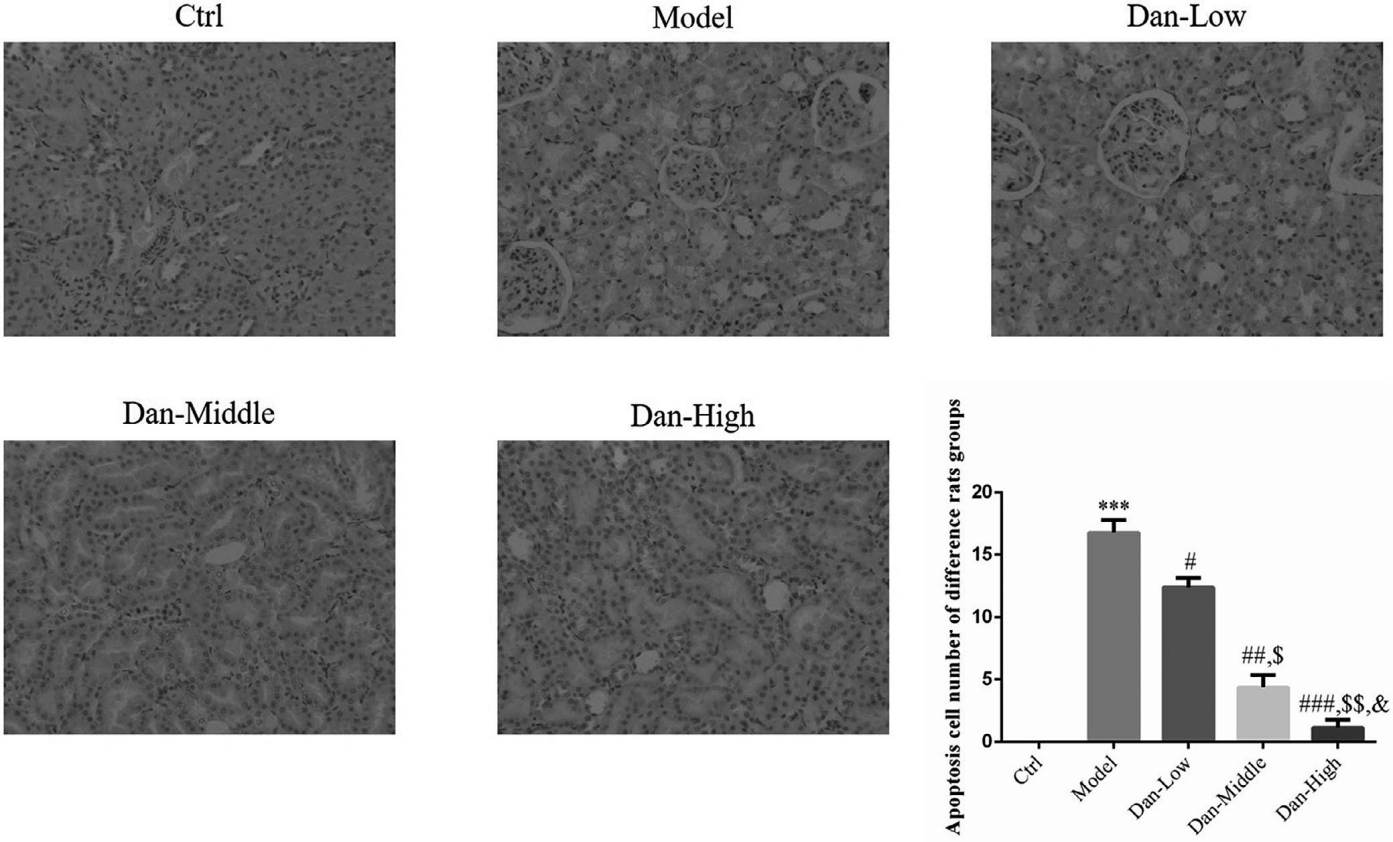
Apoptosis cell number of difference rats groups by TUNEL assay (200×). Ctrl: The rats were treated normally; Model: The rats were given DN model treatment; Dan‐Low: DN model rats treated with 10 mg/kg•day Dan; Dan‐Middle: DN model rats treated with 20 mg/kg•day Dan; Dan‐High: DN model rats treated with 100 mg/kg•day Dan. ***: *p* < .001, compared with Ctrl group; #: *p* < .05, ##: *p* < .01, ###: *p* < .001, compared with Model group; $: *p* < .05, $$: *p* < .01, compared with Dan‐Low; &: *p* < .05, compared with Dan‐Middle group

Compared with Ctrl group, the apoptosis cell number of Model group was significantly increased (*p* < .001, Figure 2). With Dan supplement, the apoptosis cell numbers were significantly depressed compared with Model group (*p* < .05, respectively, Figure 2). The apoptosis cell number were dose dependent in Dan treated groups (*p* < .05, respectively, Figure 2).

### 3.5 Protein expression of TLR4 and NF‐κB (p65) in renal tissues among the groups

**FIGURE 3:**
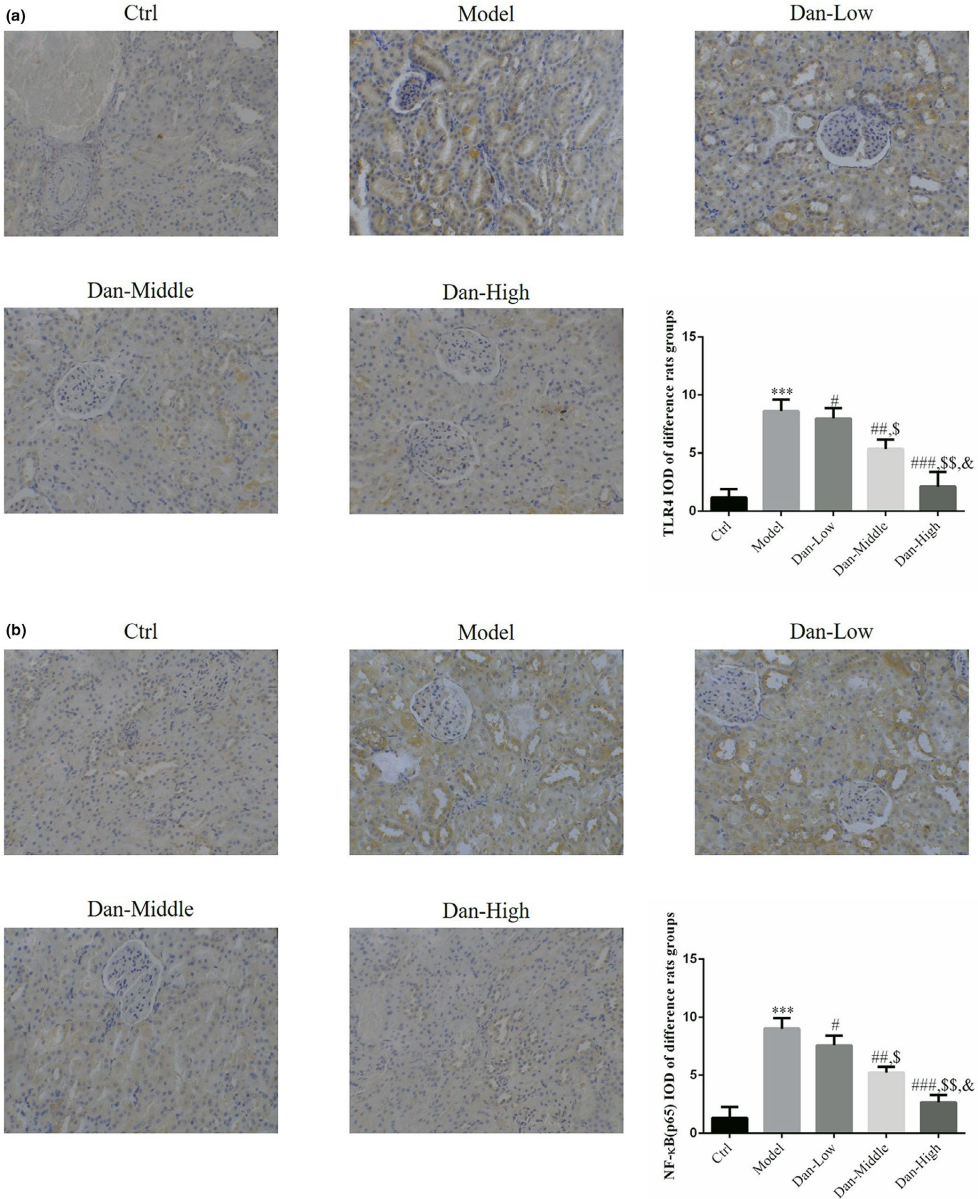
Protein expression of TLR4 and NF‐κB (p65) in renal tissues among the groups by IHC assay (200×). Ctrl: The rats were treated normally; Model: The rats were given DN model treatment; Dan‐Low: DN model rats treated with 10 mg/kg•day Dan; Dan‐Middle: DN model rats treated with 20 mg/kg•day Dan; Dan‐High: DN model rats treated with 100 mg/kg•day Dan. (a) TLR4 protein expression in difference rats groups by IHC assay (200×). ***: *p* < .001, compared with Ctrl group; #: *p* < .05, ##: *p* < .01, ###: *p* < .001, compared with Model group; $: *p* < .05, $$: *p* < .01, compared with Dan‐Low; &: *p* < .05, compared with Dan‐Middle group. (b) NF‐κB(p65) protein expression in difference rats groups by IHC assay (200×). ***: *p* < .001, compared with Ctrl group; #: *p* < .05, ##: *p* < .01, ###: *p* < .001, compared with Model group; $: *p* <.05, $$: *p* < .01, compared with Dan‐Low; &: *p* < .05, compared with Dan‐Middle group

Compared with the Ctrl group findings, the protein levels of TLR4 and NF‐κB (p65) were significantly elevated in the Model group (both *p* < .001, Figure 3a–b). After treatment with Dandelion sterol, the protein levels of both TLR4 and NF‐κB (p65) were significantly reduced compared with the findings in the Model group (both *p* < .05, Figure 3a–b), and a significant dose–effect relationship was noted among the experimental groups.

### 3.6 Related gene expression levels in rat renal tissues

**FIGURE 4:**
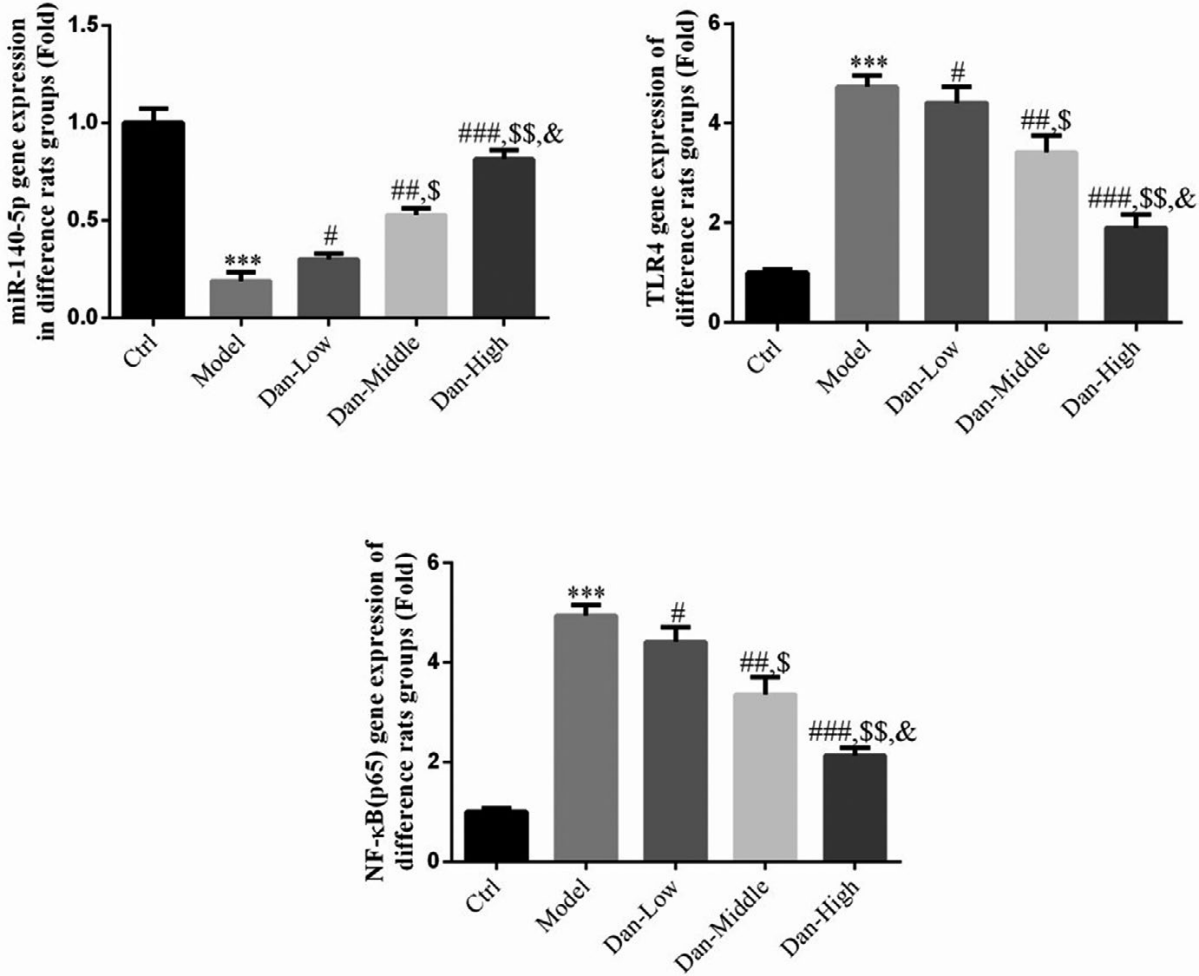
Related gene expression levels in rat renal tissues. Ctrl: The rats were treated normally; Model: The rats given DN model treatment; Dan‐Low: DN model rats treated with 10 mg/kg•day Dan; Dan‐Middle: DN model rats treated with 20 mg/kg•day Dan; Dan‐High: DN model rats treated with 100 mg/kg•day Dan. ***: *p* < .001, compared with Ctrl group; #: *p* < .05, ##: *p* < .01, ###: *p* < .001, compared with Model group; $: *p* < .05, $$: *p* < .01, compared with Dan‐Low; &: *p* < .05, compared with Dan‐Middle group

Compared with the results in Ctrl group, miR‐140‐5p gene expression was significantly decreased in the Model group, whereas the gene expression of TLR4 and NF‐κB (p65) was significantly increased (both *p* < .001, Figure 4). After treatment with Dandelion sterol, significantly reduced rates of apoptosis were observed compared with the results in the Model group (all *p* < .05, Figure 4), and there was a significant dose–effect relationship among the experimental groups (*p* < .05, Figure 4).

### 3.7 Effect of Dandelion sterol on changes in cytokine levels induced by high glucose exposure

The results of ELISA illustrated TNF‐α, IL‐1β, and IL‐6 concentrations were significantly higher in the Model group than in the Ctrl group (all *p* < .05), whereas their levels were significantly lower in the Dan‐L, Dan‐M, and Dan‐H groups than in the Model group (Table 3).

**TABLE 3:** Effect of taraxasterol on changes in cytokine levels induced by high‐glucose exposure

Group	TNF‐α(pg/ml)	IL−1β(pg/ml)	IL−6(pg/ml)
Ctrl	83.44 ± 5.71	250.15 ± 37.43	140.47 ± 15.58
Model	136.43 ± 26.55^a^	544.67 ± 75.25^a^	257.42 ± 24.33^a^
Dan‐Low	104.48 ± 16.72^a^, ^b^	364.54 ± 18.19^a^, ^b^	204.57 ± 44.14^a^, ^b^
Dan‐Middle	96.54 ± 18.75^a^, ^b^	297.46 ± 25.69^a^, ^b^	189.45 ± 28.47^a^, ^b^
Dan‐High	90.56 ± 14.65^a^, ^b^	267.67 ± 35.96^a^, ^b^	160.32 ± 26.78^a^, ^b^

^a^ *p* < .05, compared with Ctrl group.

^b^ *p* < .05, compared with Model group.

### 3.8 Effects of Dandelion sterol on apoptosis

**FIGURE 5:**
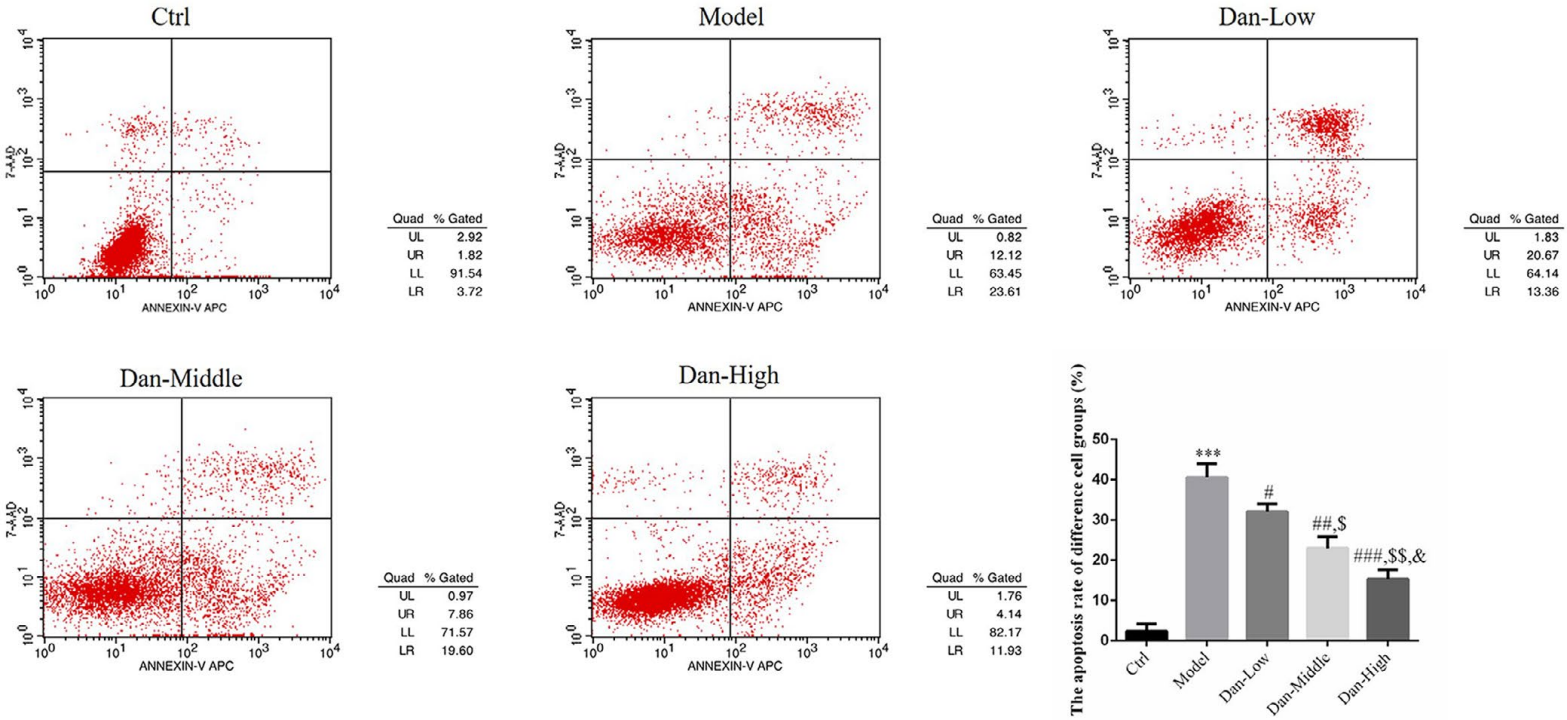
Effects of Dandelion sterol on apoptosis. Ctrl: HK‐2 cells were treated normally; Model: HK‐2 cells were treated with high glucose; Dan‐Low: HK‐2 induced by high glucose treated with 10 mg/L Dan; Dan‐Middle: HK‐2 induced by high glucose treated with 20 mg/L Dan; Dan‐High: HK‐2 induced by high glucose treated with 100 mg/L Dan. ***: *p* < .001, compared with Ctrl group; #: *p* < .05, ##: *p* < .01, ###: *p* < .001, compared with Model group; $: *p* < .05, $$: *p* < .01, compared with Dan‐Low; &: *p* < .05, compared with Dan‐Middle group

Compared with the results in the Ctrl group, a significantly higher rate of apoptosis was found in the Model group (*p* < .001, Figure 5), and these effects were reversed by treatment with Dandelion sterol (all *p* < .05, Figure 5) with a significant dose–effect relationship (*p* < .05, Figure 5).

### 3.9 Effect of Dandelion sterol on related protein expression

**FIGURE 6:**
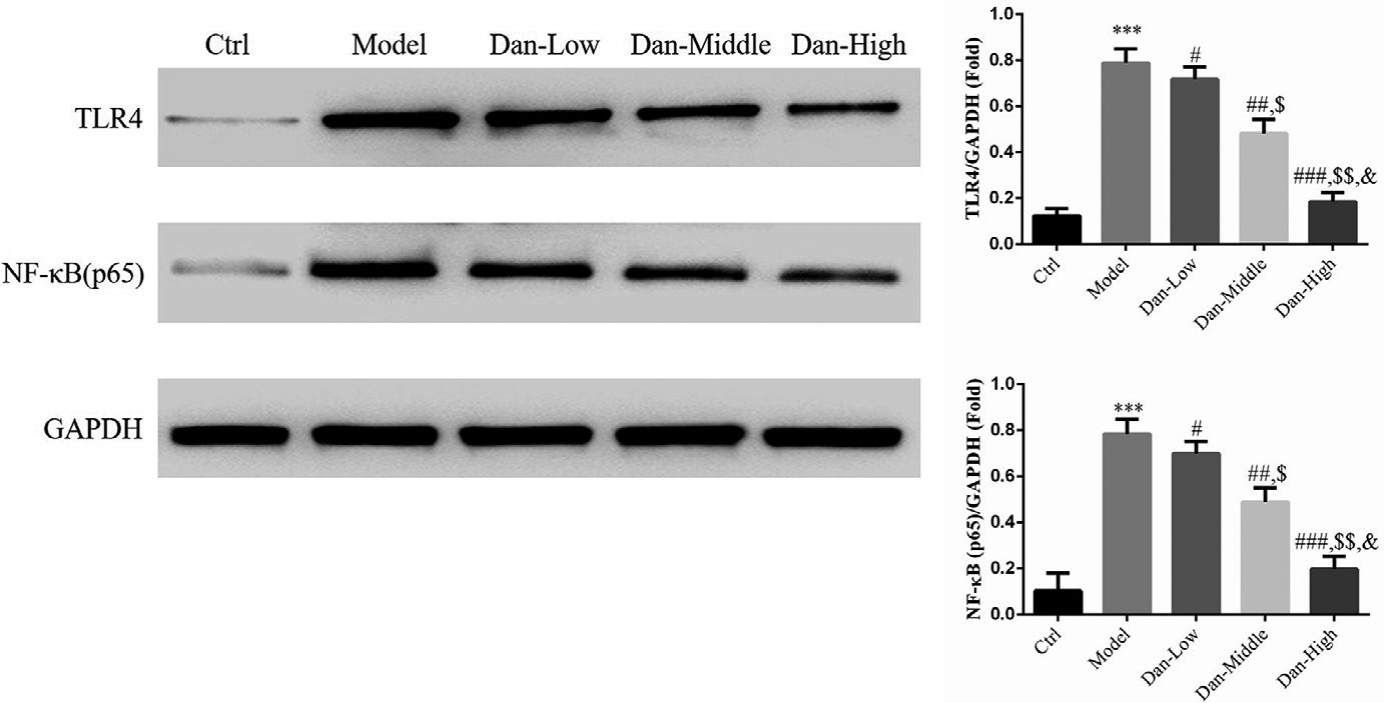
Effect of Dandelion sterol on related protein expression. Ctrl: HK‐2 cells were treated normally; Model: HK‐2 cells were treated with high glucose; Dan‐Low: HK‐2 induced by high glucose treated with 10 mg/L Dan; Dan‐Middle: HK‐2 induced by high glucose treated with 20 mg/L Dan; Dan‐High: HK‐2 induced by high glucose treated with 100 mg/L Dan. ***: *p* < .001, compared with Ctrl group; #: *p* < .05, ##: *p* < .01, ###: *p* < .001, compared with Model group; $: *p* < .05, $$: *p* < .01, compared with Dan‐Low; &: *p* < .05, compared with Dan‐Middle group

Compared with the results in the Ctrl group, TLR4 and NF‐κB (p65) protein levels were significantly increased in the Model group (both *p* < .001, Figure 6). Conversely, their expression was significantly reduced by treatment with Dandelion sterol compared with the findings in the Model group (all *p* < .05, Figure 6), and a significant dose–effect relationship was identified (*p* < .05, Figure 6).

### 3.10 Effect of Dandelion sterol on the expression of related genes

**FIGURE 7:**
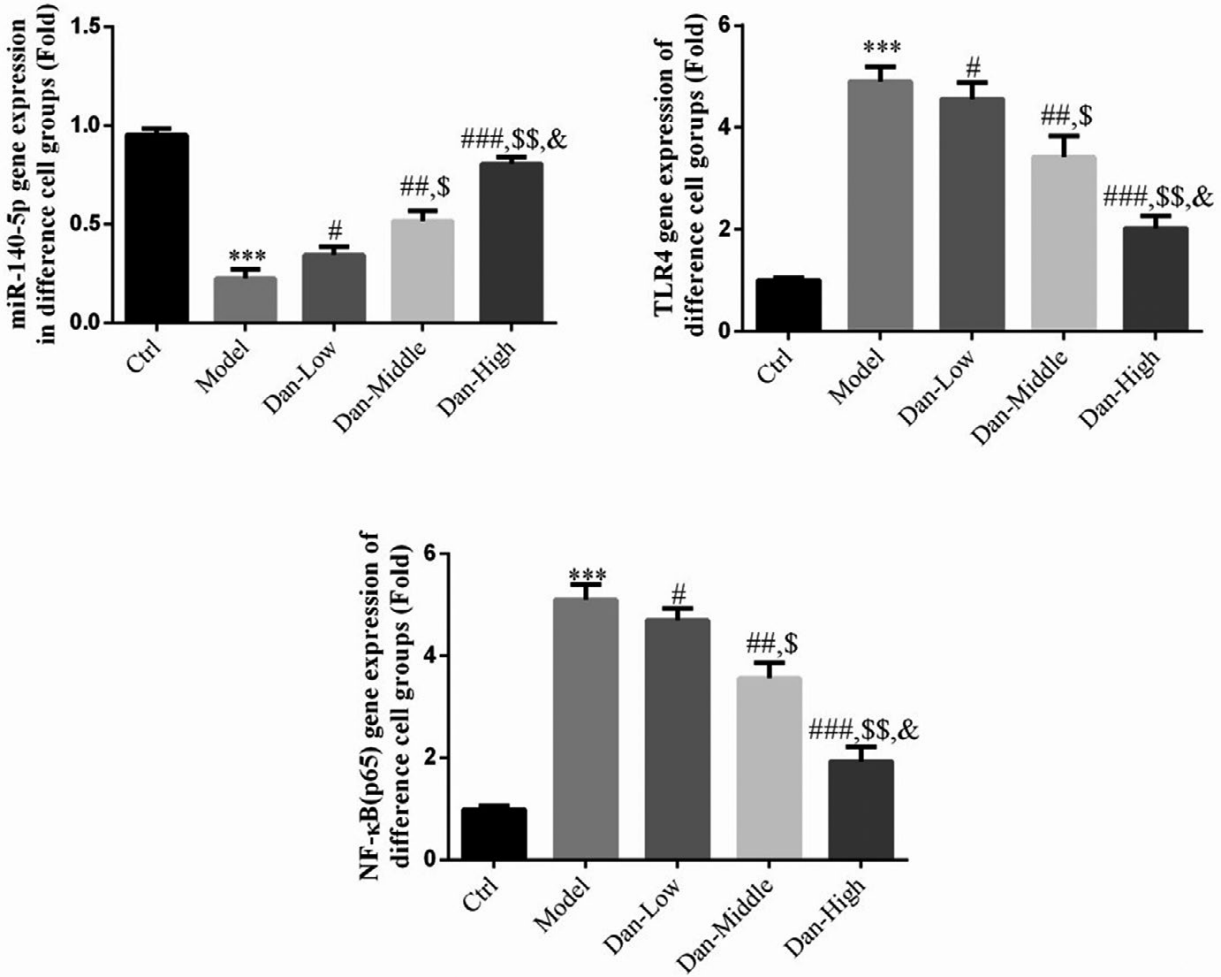
Effect of Dandelion sterol on the expression of related genes. Ctrl: HK‐2 cells were treated normally; Model: HK‐2 cells were treated with high glucose; Dan‐Low: HK‐2 induced by high glucose treated with 10 mg/L Dan; Dan‐Middle: HK‐2 induced by high glucose treated with 20 mg/L Dan; Dan‐High: HK‐2 induced by high glucose treated with 100 mg/L Dan. ***: *p* < .001, compared with Ctrl group; #: *p* < .05, ##: *p* < .01, ###: *p* < .001, compared with Model group; $: *p* < .05, $$: *p* < .01, compared with Dan‐Low; &: *p* < .05, compared with Dan‐Middle group

Compared with the findings in the Ctrl group, TLR4 and NF‐κB (p65) gene expression was significantly increased and miR‐140‐5p gene expression was significantly reduced in the Model group (all *p* < .001, Figure 7). After treatment with Dandelion sterol, significant downregulation of TLR4 and NF‐κB (p65) and significant upregulation of miR‐140‐5p were observed compared with the levels in the Model group (all *p* < .05, Figure 7), exhibiting a significant dose–effect relationship (*p* < .05, Figure 7).

### 3.11 Effect of Dandelion sterol on nuclear NF‐κB (p65) protein levels

**FIGURE 8:**
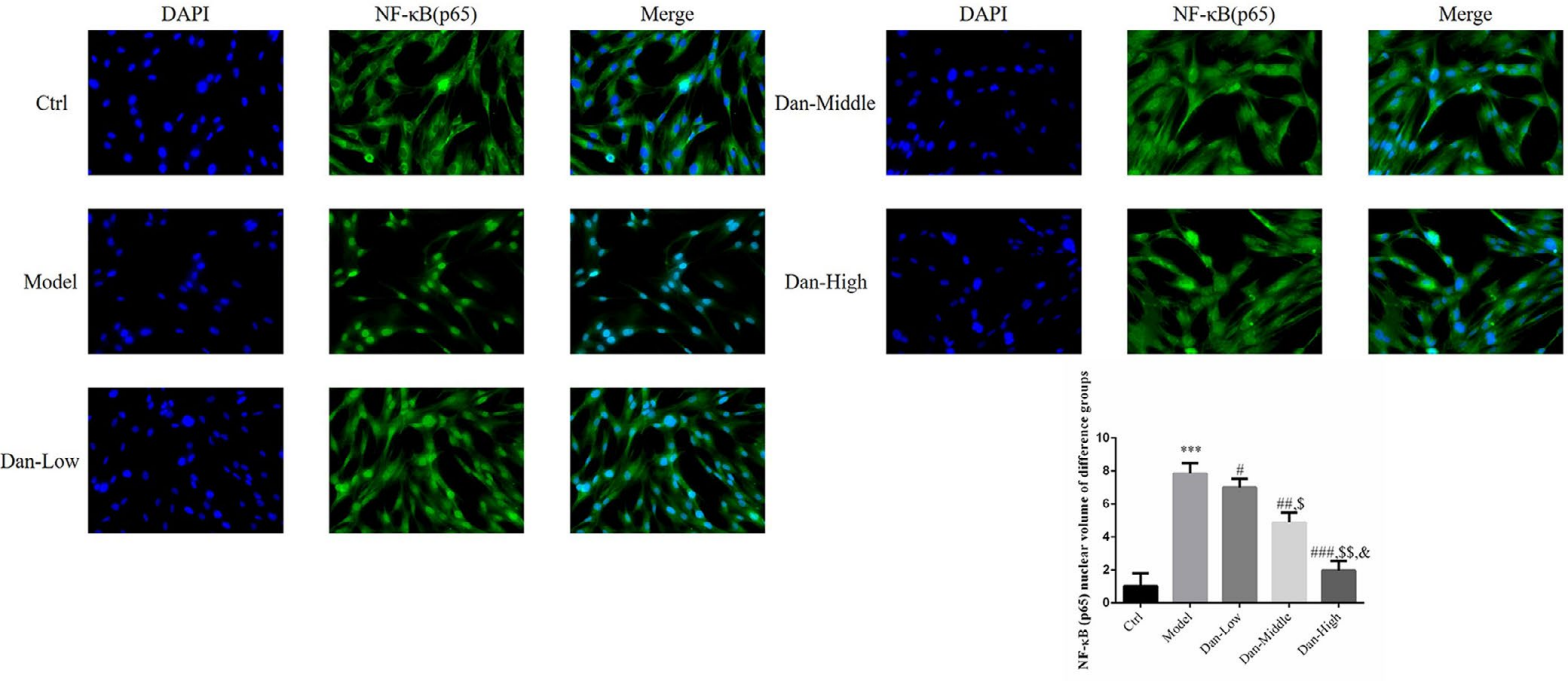
Effect of Dandelion sterol on nuclear NF‐κB (p65) protein levels. Ctrl: HK‐2 cells were treated normally; Model: HK‐2 cells were treated with high glucose; Dan‐Low: HK‐2 induced by high glucose treated with 10 mg/L Dan; Dan‐Middle: HK‐2 induced by high glucose treated with 20 mg/L Dan; Dan‐High: HK‐2 induced by high glucose treated with 100 mg/L Dan. ***: *p* < .001, compared with Ctrl group; #: *p* < .05, ##: *p* < .01, ###: *p* < .001, compared with Model group; $: *p* < .05, $$: *p* < .01, compared with Dan‐Low; &: *p* < .05, compared with Dan‐Middle group

Compared with the findings in the Ctrl group, significantly increased NF‐κB (p65) translocation to the nucleus was observed in the Model group (*p* < .001, Figure 8). This finding was significantly reversed in the Dan‐L, Dan‐M, and Dan‐H groups (all *p* < .05, Figure 8), and a significant dose–effect relationship was noted (*p* < .05, Figure 8).

Influence of miR‐140‐5p on the effects of Dandelion sterol on the improvement of inflammatory factor expression in high‐glucose–exposed HK‐2 cells.

Compared with the results in the Ctrl group, TNF‐α, IL‐1β, and IL‐6 levels were significantly increased in the Model group (all *p* < .05, Table 4). Their levels were all significantly decreased by treatment with Dandelion sterol compared with those in the Model group (all *p* < .05, Table 4). The effects of Dandelion sterol on TNF‐α, IL‐1β, and IL‐6 levels were abrogated by the simultaneous suppression of miR‐140‐5p (all *p* < .05, Table 4).

**TABLE 4:** Influence of miR‐140‐5p on the effects of taraxasterol on the improvement of inflammatory factor expression in high‐glucose–exposed HK‐2 cells

Group	TNF‐α(pg/ml)	IL−1β(pg/ml)	IL−6(pg/ml)
Ctrl	83.44 ± 5.71	250.15 ± 37.43	140.47 ± 15.58
Model	136.43 ± 26.55^a^	544.67 ± 75.25^a^	257.42 ± 24.33^a^
miR inhibitor‐NC	135.67 ± 27.84^a^	547.58 ± 78.44^a^	256.98 ± 25.54^a^
Dan	93.46 ± 15.66^a^, ^b^	268.57 ± 36.07^a^, ^b^	165.38 ± 27.58^a^, ^b^
Dan+miR inhibitor	138.53 ± 27.35^c^	539.47 ± 76.51^c^	255.72 ± 26.38^c^

^a^ *p* < .05, compared with Ctrl group.

^b^ *p* < .05, compared with Model group.

^c^ *p* < .05, compared with Dan group.

### 3.12 Influence of miR‐140‐5p on the effects of Dandelion sterol on the improvement of high‐glucose–induced HK‐2 cell apoptosis

**FIGURE 9:**
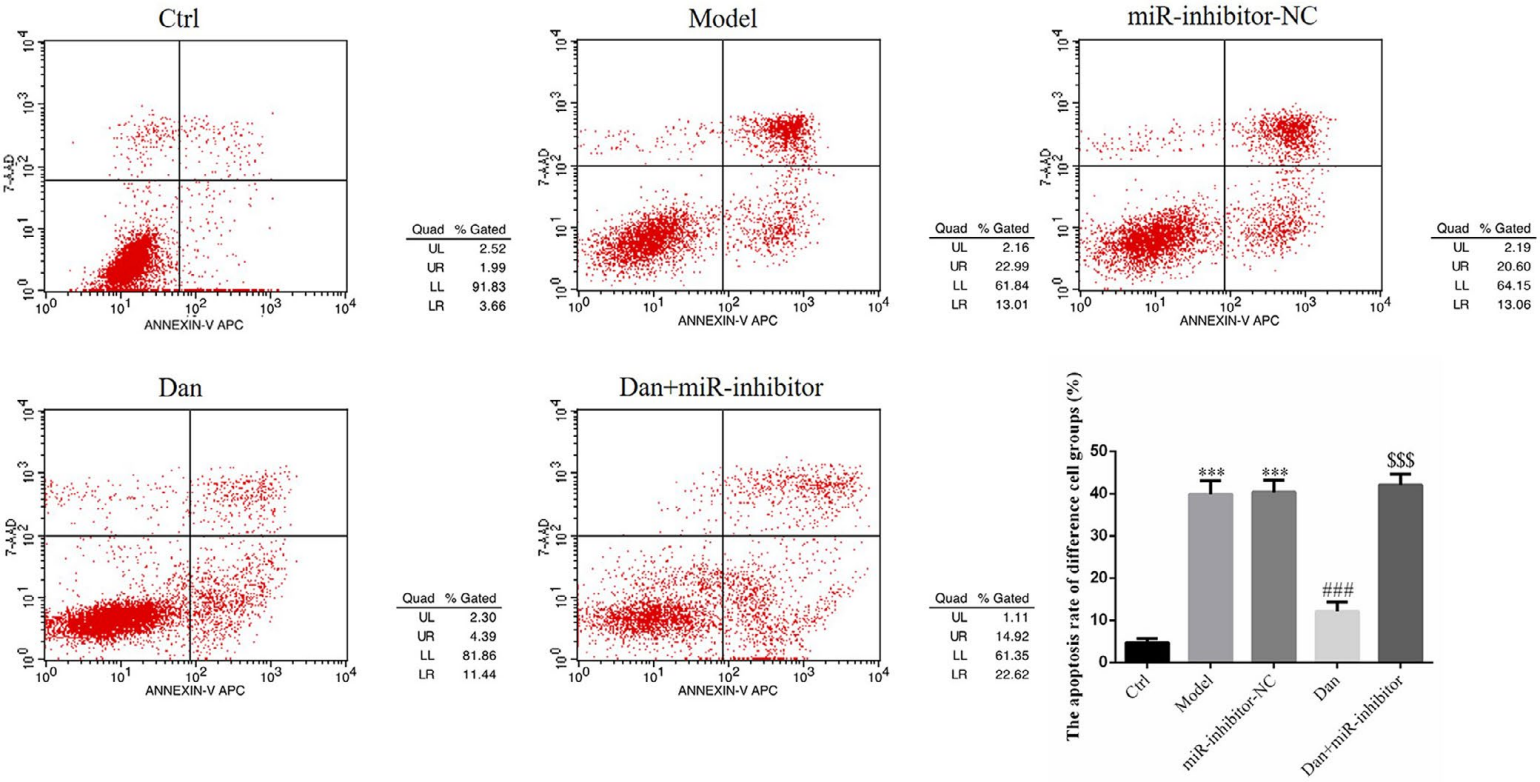
Influence of miR‐140‐5p on the effects of Dandelion sterol on the improvement of high‐glucose–induced HK‐2 cell apoptosis. Ctrl: HK‐2 cells were treated normally; Model: HK‐2 cells were treated with high glucose; miR‐inhibitor‐NC: HK‐2 induced by high glucose and transfected with miR‐inhibitor‐NC; Dan: HK‐2 induced by high glucose treated with 100 mg/L Dan; Dan+miR‐inhibitor: HK‐2 induced by high glucose treated with 100 mg/L Dan and transfected with miR inhibitor. ***: *p* < .001, compared with Ctrl; ###: *p* < .001, compared with Model group; $$$: *p* < .001, compared with Dan

Compared with the findings in the Ctrl group, apoptosis rates were significantly higher in the Model group and miR‐inhibitor‐NC groups (both *p* < .001, Figure 9), and no significant difference in the apoptosis rate was found between the Model and miR‐inhibitor‐NC groups. Apoptosis was significantly inhibited by treatment with Dandelion sterol compared with the findings in the Model group (all *p* < .001, Figure 9). Meanwhile, the effects of Dandelion sterol on apoptosis were blocked by the suppression of miR‐140‐5p (all *p* < .001, Figure 9).

### 3.13 Influence of miR‐140‐5p on the effects of Dandelion sterol on protein expression in high‐glucose–treated HK‐2 cells

**FIGURE 10:**
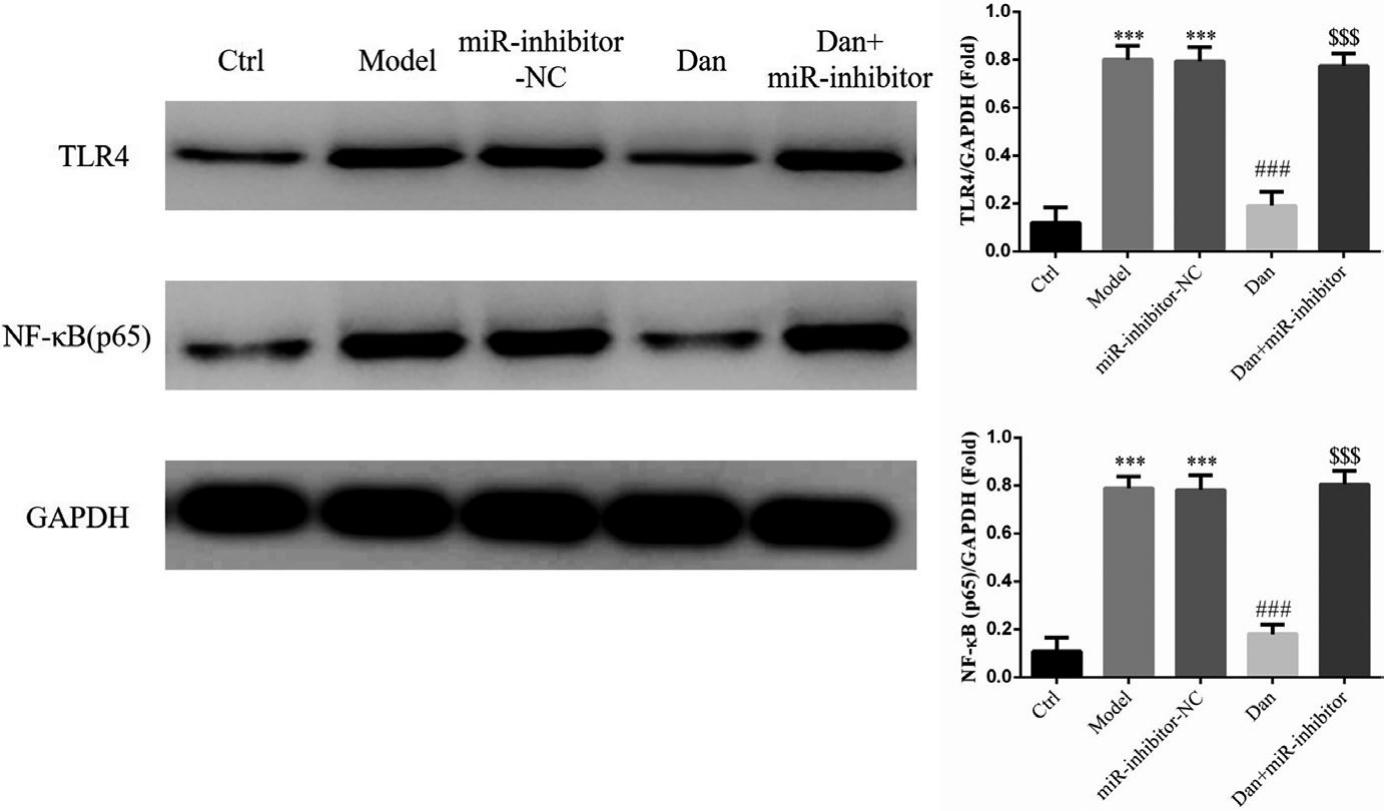
Influence of miR‐140‐5p on the effects of Dandelion sterol on protein expression in high‐glucose–treated HK‐2 cells. Ctrl: HK‐2 cells were treated normally; Model: HK‐2 cells were treated with high glucose; miR‐inhibitor‐NC: HK‐2 induced by high glucose and transfected with miR‐inhibitor‐NC; Dan: HK‐2 induced by high glucose treated with 100 mg/L Dan; Dan+miR‐inhibitor: HK‐2 induced by high glucose treated with 100 mg/L Dan and transfected with miR inhibitor. ***: *p* < .001, compared with Ctrl; ###: *p* < .001, compared with Model group; $$$: *p* < .001, compared with Dan

Compared with the Ctrl group findings, TLR4 and NF‐κB (p65) protein levels were significantly elevated in the Model and miR‐inhibitor‐NC groups (all *p* < .001, Figure 10), and no significant difference was noted between the Model and miR‐inhibitor‐NC groups. Compared with the results in the Model group, TLR4 and NF‐κB (p65) protein expression were significantly inhibited in the Dan‐L, Dan‐M, and Dan‐H groups (all *p* < .001, Figure 10), and these effects of Dandelion sterol were abrogated by the suppression of miR‐140‐5p (all *p* < .001, Figure 10).

### 3.14 Influence of miR‐140‐5p on the effects of Dandelion sterol on related gene expression in high‐glucose–treated HK‐2 cells

**FIGURE 11:**
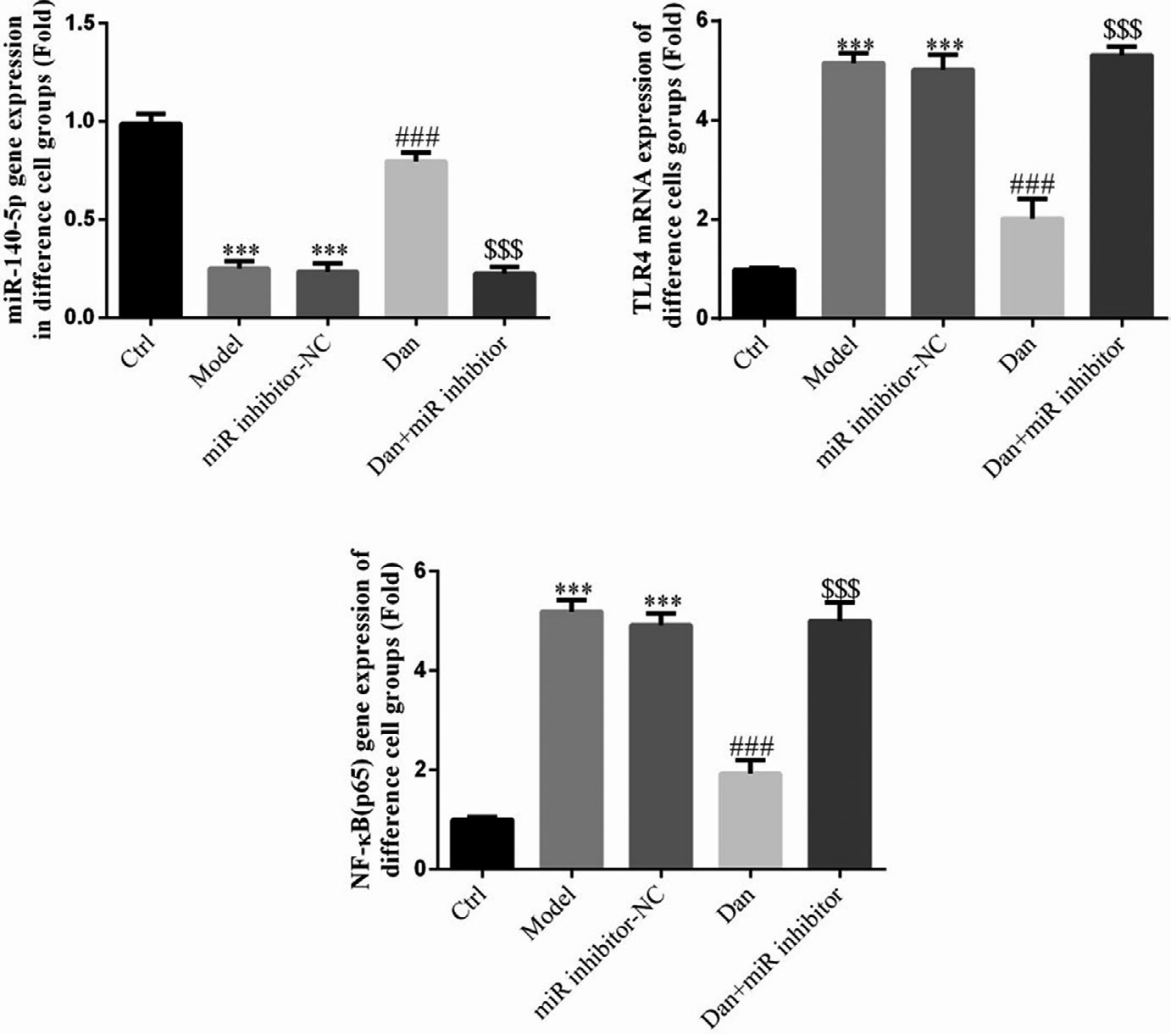
Influence of miR‐140‐5p on the effects of Dandelion sterol on related gene expression in high‐glucose–treated HK‐2 cells. Ctrl: HK‐2 cells were treated normally; Model: HK‐2 cells were treated with high glucose; miR‐inhibitor‐NC: HK‐2 induced by high glucose and transfected with miR‐inhibitor‐NC; Dan: HK‐2 induced by high glucose treated with 100 mg/L Dan; Dan+miR‐inhibitor: HK‐2 induced by high glucose treated with 100 mg/L Dan and transfected with miR inhibitor. ***: *p* < .001, compared with Ctrl; ###: *p* < .001, compared with Model group; $$$: *p* < .001, compared with Dan

Compared with the findings in the Ctrl group, TLR4 and NF‐κB (p65) gene expression were significantly increased in the Model and miR‐inhibitor‐NC groups, whereas miR‐140‐5p gene expression was significantly decreased in these groups (all *p* < .001, Figure 11). No significant difference in gene expression was observed between the Model and miR‐inhibitor‐NC groups. Compared with the results in the Model group, TLR4 and NF‐κB (p65) levels were significantly decreased by treatment with Dandelion sterol, whereas miR‐140‐5p gene expression was significantly increased (all *p* < .001, Figure 11). Meanwhile, simultaneous suppression of miR‐140‐5p and treatment with Dandelion sterol resulted in significantly increased TLR4 and NF‐κB (p65) levels and significantly reduced miR‐140‐5p levels compared with the results in cells treated with Dandelion sterol alone (all *p* < .001, Figure 11).

### 3.15 Influence of miR‐140‐5p on the effects of Dandelion sterol on nuclear NF‐κB(p65) accumulation in high‐glucose–treated HK‐2 cells

**FIGURE 12:**
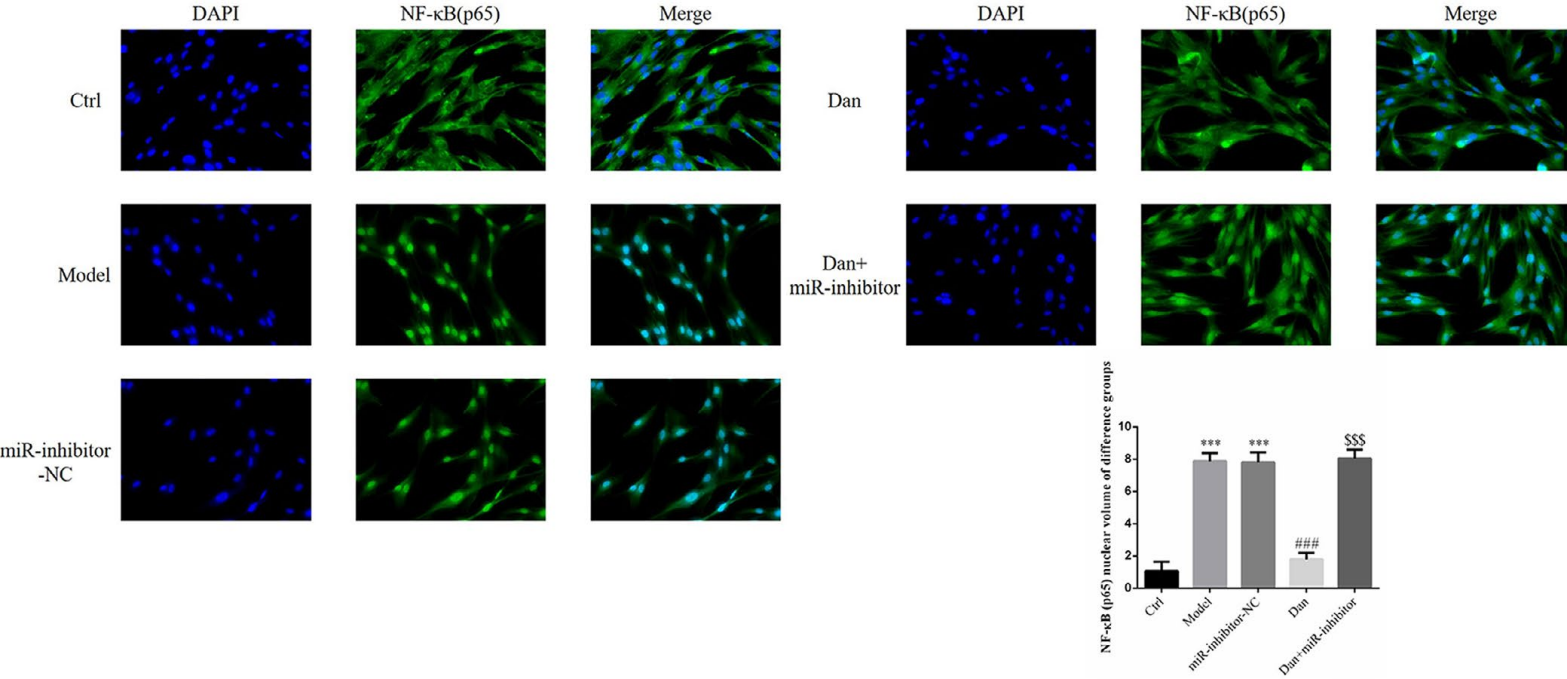
Influence of miR‐140‐5p on the effects of Dandelion sterol on nuclear NF‐κB(p65) accumulation in high‐glucose–treated HK‐2 cells. Ctrl: HK‐2 cells were treated normally; Model: HK‐2 cells were treated with high glucose; miR‐inhibitor‐NC: HK‐2 induced by high glucose and transfected with miR‐inhibitor‐NC; Dan: HK‐2 induced by high glucose treated with 100 mg/L Dan; Dan+miR‐inhibitor: HK‐2 induced by high glucose treated with 100 mg/L Dan and transfected with miR inhibitor. ***: *p* < .001, compared with Ctrl; ###: *p* < .001, compared with Model group; $$$: *p* < .001, compared with Dan

Compared with the findings in the Ctrl group, we observed significantly increased nuclear accumulation of NF‐κB (p65) protein in the Model and miR‐inhibitor‐NC groups (both *p* < .001, Figure 12), and no significant difference in nuclear NF‐κB (p65) protein levels was noted between these two groups. Conversely, nuclear NF‐κB (p65) levels were significantly decreased by treatment with Dandelion sterol (all *p* < .001, Figure 12), but these effects of Dandelion sterol were abrogated by the simultaneous suppression of miR‐140‐5p (all *p* < .001, Figure 12).

## 4 DISCUSSION

It has been reported that high blood sugar levels as a result of the long‐term failure of blood glucose control, as a marker of DM, can cause damage to organs and may potentially result in life‐threatening complications, such as cardiovascular, neurogenic, and eye and kidney diseases. Among the DM‐related complications, the incidence of DN has increased, affecting most patients with DM (Thomas et al., 2016). DN is mainly characterized by the loss of renal function, which further leads to renal tissue damage. The pathogenesis of renal injury in DN is complex, and oxidative stress injury, immune inflammatory responses, mitochondria‐related apoptosis, and other factors were demonstrated to interact with each other and play crucial roles in the pathogenesis of renal injury in DN (Eriguchi et al., 2018; Sifuentes‐Franco et al., 2018). Prior studies illustrated that TLR4, a pattern recognition receptor, can recognize endogenous ligands and then induce signal transduction in response to high blood sugar levels, playing an important role in the progression of renal injury in DN (Cha et al., 2013; Lin & Tang, 2014). In this study, as confirmed by in vivo and in vitro experiments, TLR4 and NF‐κB (p65) were activated in rodent models of DM, resulting in increased apoptosis. DN‐induced renal injury was significantly improved by Dandelion sterol, which was also associated with significantly reduced apoptosis, downregulation of TLR4 and NF‐κB (p65), and reduced nuclear accumulation of NF‐κB (p65).

In general, dandelion is used as a nontoxic natural medicine with multiple biological activities, and dandelion sterol has been widely used as a dandelion extract. Additionally, it has been reported that dandelion sterol exhibits antiinflammatory effects in animal models (Wang et al., 2016), and it could inhibit oxidative damage‐induced lung inflammation by regulating TLR4 expression (Xueshibojie et al., 2016). Moreover, as suggested by a previous study, dandelion sterol can exert protective effects against apoptosis in H2O2‐induced human umbilical vein endothelial cell injury by inhibiting the expression of vascular cell adhesion molecule 1 and cluster of differentiation 80. Furthermore, dandelion sterol was reported to exert protective effects on lipopolysaccharide‐damaged macrophages by inhibiting the expression of inducible nitric oxide synthase and cyclooxygenase‐2 via the regulation of the ERKl/2 and p38 signaling pathways (Xiong et al., 2014). To explore the mechanism by which Dandelion sterol regulates TLR4/NF‐κB (p65) signaling, the related miRNAs were analyzed in the study.

Several studies have revealed that the regulation of related miRNAs can effectively improve DN‐induced renal injury (Chen et al., 2014; Kaidonis et al., 2016; Yang et al., 2018; Yao et al., 2018). In addition, it has been reported that miR‐140‐5p has a targeted inhibitory effect on TLR4, thereby regulating the inflammatory response caused by TLR4/NF‐κB (p65) activation (Yang et al., 2018; Zhang et al., 2020). In this study, we found that the gene expression of miR‐140‐5p was significantly increased by treatment with Dandelion sterol, and an obvious dose–effect relationship was observed. The cell experiments demonstrated that the effects of Dandelion sterol were significantly suppressed by the transfection of an miR‐140‐5p inhibitor into HK‐2 cells. This result revealed that the beneficial effects of Dandelion sterol on DM‐induced renal injury are closely related to the upregulation of miR‐140‐5p.

In conclusion, our in vivo and in vitro experiments confirmed that dandelion sterol can improve DM‐induced damage to renal tissue cells by inhibiting the TLR4/NF‐κB (p65) signaling pathway through the regulation of miR‐140‐5p.

## AUTHOR CONTRIBUTIONS

**Lin Tian:** Data curation (equal); Formal analysis (equal); Resources (equal); Software (equal). **Peng Fu:** Investigation (equal); Methodology (equal); Visualization (equal); Writing‐original draft (equal); Writing‐review & editing (equal). **Min Zhou:** Formal analysis (equal); Investigation (equal); Visualization (equal); Writing‐original draft (equal). **Jiping Qi:** Investigation (equal); Methodology (equal); Software (equal); Supervision (equal); Visualization (equal).

## Data Availability

None.
